# Long-term outcomes of colectomy surgery among patients with ulcerative colitis

**DOI:** 10.1186/s40064-015-1350-7

**Published:** 2015-10-05

**Authors:** Carl Brown, Peter R. Gibson, Ailsa Hart, Gilaad G. Kaplan, Sumesh Kachroo, Qian Ding, Emily Hautamaki, Tao Fan, Christopher M. Black, Xiaohan Hu, Kathleen Beusterien

**Affiliations:** Division of General Surgery, Providence Health Care, St. Paul’s Hospital, Room C310, St Paul’s Hospital, 1081 Burrard Street, Vancouver, BC V6Z 1Y6 Canada; Department of Gastroenterology, The Alfred Hospital and Monash University, Melbourne, VIC 3004 Australia; IBD Unit, St. Mark’s Hospital NWLH NHS Trust, Northwick Park, Harrow, London HA1 3UJ UK; Departments of Medicine and Community Health Sciences, University of Calgary, Calgary, AB T2N 4N1 Canada; Merck & Co. Inc., Rahway, 07065 NJ USA; Ferris University, Big Rapids, 49307 MI USA; Oxford Outcomes Inc., an ICON plc company, Bethesda, 20814 MD USA; Sanofi US and Center for Clinical Epidemiology and Biostatistics, Bridgewater, 08807 NJ USA; University of Pennsylvania School of Medicine, Philadelphia, 19104 NJ USA; Outcomes Research Strategies in Health, Washington, 20008 DC USA

**Keywords:** Ulcerative colitis, Colectomy, Survey, Quality of life, pouchitis/pouch failure

## Abstract

The objective of this study was to evaluate long-term health-related quality of life outcomes among patients who had a colectomy within the previous 10 years. A cross-sectional survey was administered to consecutive patients ≥18 years of age with ulcerative colitis who had a colectomy within the last 10 years from centers in Canada, Australia, and the United Kingdom. Data were extracted from medical chart reviews to confirm selected self-reported patient characteristics. Of 351 survey respondents, 49 % were male and the median age was 40 years (interquartile range 30–52). Respondents were diagnosed with UC a median of 9.2 (5.7–15.1) years prior to the survey and first surgery occurred a median of 3.7 (2.1–5.8) years ago. Although most respondents (84 %) reported improved quality of life compared to the status before surgery, 81 % experienced problems in at least one of the following areas: depression, work productivity, restrictions in diet, body image, and sexual function. According to HADS scores, 30 and 17 % of survey respondents experienced anxiety and depression, respectively. Among moderate to severe UC patients pre-colectomy, 27 % of men and 28 % of women reported that their sexual life was worse now than before surgery. The mean EQ-5D utility index score overall was 0.79 (95 % confidence interval 0.77–0.81). Quality of life after colectomy for UC is generally good, but there are persistent quality of life issues that impact multiple domains, including psychological and sexual functioning.

**Electronic supplementary material:**

The online version of this article (doi:10.1186/s40064-015-1350-7) contains supplementary material, which is available to authorized users.

## Background

Ulcerative colitis (UC) is an inflammatory bowel disease (IBD) characterized by chronically active or recurrent episodes of inflammation of the colon. It is estimated to affect approximately 0.25 % of the general population in the Western world (Gracie and Ford 2012; Molodecky et al. 2012). Signs and symptoms of UC, encompassing rectal bleeding, bloody diarrhea, fecal incontinence, abdominal cramps and pain, range from mild to severe and can substantially impact health-related quality of life (HRQL) (Schreiber et al. 2012). When pharmacological/biological management of UC has failed or in the context of neoplasia, colectomy is indicated. The 10-year risk of colectomy following diagnosis of ulcerative colitis is 16 % (Frolkis et al. 2013). The current gold standard surgery for UC patients is proctocolectomy with an ileal pouch anal anastomosis (IPAA) (Travis et al. 2008).

Although proctocolectomy has many advantages, it does have disadvantages, especially with respect to clinical and humanistic burden. Post-proctocolectomy complications include pouchitis, pouch leakage, pelvic abscesses, pouch fistulae, small bowel obstruction, anastomotic stricture, post-operative bleeding, faecal incontinence, sexual dysfunction, infections, delayed wound healing, and nerve damage (Frolkis et al. 2014; de Silva et al. 2011; Dayan and Turner 2012; Kaplan et al. 2008; Hueting et al. 2004, 2005; Lichtenstein et al. 2006).

A retrospective analysis of 666 patients with UC who underwent a colectomy reported that 27 % experienced severe postoperative complications, with the mortality rate being 1.5 % (de Silva et al. 2011). This study also highlighted that elderly patients and patients with multiple comorbidities are more likely to experience complications. A meta-analysis demonstrated that the risk of postoperative mortality is 0.7 % following an elective operation and 5.3 % following an emergent colectomy for UC (Singh et al. 2014). Another meta-analysis showed an approximate threefold increase (from 15 to 48 %) in the risk of infertility in women with UC as a result of IPAA (Waljee et al. 2006). Johnson et al. reported the infertility rate in females who had pelvic pouch surgery was significantly higher as compared to females who were managed medically (38.1 % compared with 13.3 %; p < 0.001) (Johnson et al. 2004).

Published studies that have evaluated HRQL among UC patients after proctocolectomy vary in their conclusions, with some reporting significant improvement and others modest or no improvement and with certain negative long-term consequences (Berndtsson and Oresland 2003; Fazio et al. 1999; Martin et al. 1998; Van Balkom et al. 2012; Fazio et al. 2013). A systematic review identifying 33 studies describing QoL, HRQL and health status of UC patients after IPAA found that although HRQL and health status were generally improved 12 months after proctocolectomy with IPAA, QoL was not reported by any study (Heikens et al. 2012). Given the variability in findings across UC QoL studies, it would be useful to have a clear understanding of the effects of proctocolectomy with IPAA or ileostomy on patient QoL. Such data can help patients and physicians weigh the pros and the cons of the surgery and help provide more personalized care to these patients. This study was aimed to evaluate the long-term health-related quality of life (HRQL) outcomes of UC patients from Europe, North America, and Australia after having a proctocolectomy for UC using a variety of survey tools.

## Methods

### Study design

This study was a cross-sectional survey administered online or via paper to patients with UC who had a colectomy within the past 10 years with data collected from November 2010 to July 2011 and is referred to as the Long-term Outcomes of Colectomy Surgery among Ulcerative Colitis Patients Study (LOCUS). For descriptive purposes, this study refers to colectomy and proctocolectomy collectively as ‘colectomy’.

### Study population

The study population consisted of patients treated at five centers in Canada, Australia, and the UK. In Canada and Australia, ethics approval was obtained at site-specific institutional review boards (IRBs). In the UK, the study was approved by the National Health Service Research and Development Forum (NHS R&D) and supported by the National Institute for Health Research Comprehensive Clinical Research Network (NIHR CCRN). In addition, for UK patients recruited through a patient recruitment agency, the study was approved by a commercial IRB (MaGil IRB, Rockville, MD, USA).

Patients were included if surgery for UC occurred not greater than 10 years prior to entering the study, if they were at least 18 years of age and less than 65 years of age at the date of colectomy, and if they were fluent in English and willing to provide informed consent. Persons were excluded from participating in the study if they had Crohn’s disease or colon cancer, abdominal surgery less than 2 months prior to the date of screening, or did not have competency to provide fully informed consent in the screener’s opinion.

### Procedures

Consecutive patients who were potentially eligible were identified via databases in hospital clinics and in private practice settings of both gastroenterologists and colorectal surgeons. Those fulfilling the eligibility criteria were recruited by site coordinators who sent study participants a web link to complete the survey or mailed the paper questionnaire. A medical chart review was performed by site coordinators to confirm the diagnosis and date of diagnosis of UC, type and date of surgery and reported postoperative complications (e.g., pouchitis). This confirmation was done to minimize the risk of self-report bias and misclassification errors.

This study included the below seven survey tools.

- *Inflammatory Bowel Disease Questionnaire* (*IBDQ*) This 32-item questionnaire was used to measure bowel symptoms, systemic symptoms, and emotional and social functioning with respect to UC. The total IBDQ score, which ranges from 32 to 224 with higher scores indicating better functioning, was used to determine patients’ overall level of functioning (Guyatt et al. 1989).
- *EQ*-*5D* This health-status utility measure assesses five basic life domains (mobility, self-care, usual activities, pain/discomfort, and anxiety/depression), was used to compute utility weights. Utilities are configured such that 0.0 is associated with being dead and 1.0 is associated with full health; thus, a higher utility value is considered better (EuroQol 1990).
- *Body Image Questionnaire* (*BIQ*) This was used to assess body image and satisfaction with surgical scarring post-colectomy. Five general body image items are scored on a 4-point scale from 1 (“no, not at all”) to 4 (“yes, extremely”), and are summed to compute a score ranging from 5 to 20, with higher scores indicating poorer body image. The second set of items pertain to the colectomy surgical scar, scored on a 7-point scale from “very unsatisfied” to “very satisfied” or “revolting” to “beautiful”. Body Image Scale scores were compared to previously published scores of colectomy patients by Polle 2007, Dunker 1998, and Larson 2008 (Löwe and Clement 1996).
- *Medical Outcomes Study Sexual Functioning Scale* (*MOS*-*SFS*) This 4-item measure of sexual functioning from the RAND Corporation’s Medical Outcomes Study (MOS) has been used in a variety of patient populations and the general US population. Higher scores indicate worse sexual functioning. A “do not wish to answer” option was added for this study (patients who did not wish to answer were treated as missing in the analysis) (Tarlov et al. 1989).
- *Hospital Anxiety and Depression Scale* (*HADS*) This 14-item self-report measure designed to assess levels of anxiety and depression was used to measure general levels of mood symptomatology. Subscale scores for anxiety and depression range from 0 to 21 with lower scores indicating fewer mood-related symptoms. A cut-off of 7/8 is used to classify mild intensity and a cut-off of 10/11 is used to classify severe intensity for the anxiety and depression subscales (Zigmond and Snaith 1983).
- *Dietary Restrictions Questions* This survey included three questions related to dietary restrictions, each of which was analyzed separately.
- *WHO Work Performance Questionnaire*—*Absenteeism and Presenteeism* (*WHO*-*HPQ*-*AP*) This is a set of seven items that measures work performance by examining normal working schedule, missed work due to health-related difficulties, scheduled time off work (i.e., non-health related missed work), and general level of job performance. Better work performance is indicated by low levels of absenteeism and high levels of presenteeism (Kessler et al. 2003).

The proportions of LOCUS participants who reported detriments in the HRQL domains of mood (i.e. depression), work productivity, diet (i.e. greater eating restrictions), sexual life, body image, and ongoing need for medication for bowel condition were evaluated and reported.

### Analyses

Descriptive statistics, including mean scores and proportions, were calculated for the items and scales, as applicable. The IBDQ, EQ-5D, MOS-SFS, WHO-HPQ, and HADS were scored according to scoring instructions of the developer. EQ-5D scores were calculated using UK weights. Selected item-level and scale scores were compared using Wilcoxon–Mann–Whitney test or Chi square, as applicable. All comparisons were tested using two-tailed tests at α = 0.05. All analyses were performed using SAS Enterprise Guide Version 4.3 (Cary, NC, USA).

## Results

### Study population

**Fig. 1:**
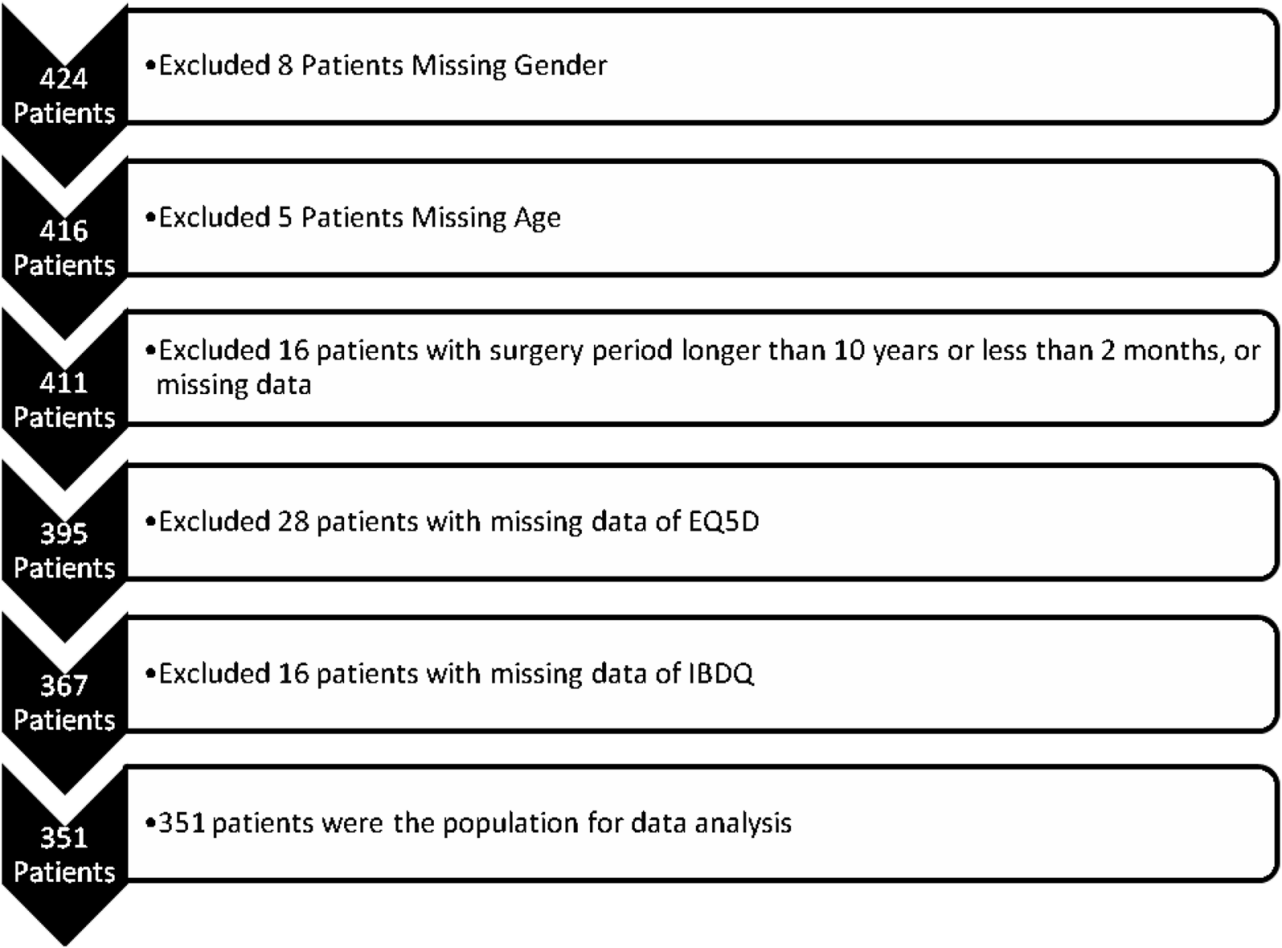
Flowchart of the Patients who were included in the statistical analysis

The surveys were sent to 743 eligible patients who met the inclusion criteria and 424 patients (57 %) returned the surveys. However, 73 patients were excluded from analysis because of incomplete key information for primary objectives in the questionnaire (Fig. 1). Thus, a total of 351 patients with complete surveys were included in the analysis, including 126 in Canada, 126 in the UK, and 99 in Australia. Demographics and clinical characteristics are presented in Table 1. Of survey respondents (n = 351) 49 % were male and the median age was 40 years (interquartile range 30–52). Respondents were diagnosed with UC a median of 9.2 (5.7–15.1) years prior to the survey and first surgery occurred a median of 3.7 (2.1–5.8) years ago. The majority of respondents reported moderate to severe UC prior to surgery. Approximately one-third (32 %) had a stoma at some time after the creation of the ileal pouch and 36 % reported a history of pouch complications.

**Table 1:** Demographics and clinical characteristics of participants

Characteristic	All	Australia	Canada	United Kingdom
Participants, n (%)	351	99 (28 %)	126 (36 %)	126 (36 %)
Gender, n (%)				
Male	173 (49 %)	50 (51 %)	59 (47 %)	64 (51 %)
Female	178 (51 %)	49 (49 %)	67 (53 %)	62 (49 %)
Age (years), median (IQR)	40 (30–52)	42 (33–53)	40 (29–52)	38 (31–49)
Years since first surgery, median (IQR)	3.7 (2.1–5.8)	3.4 (1.4–5.5)	3.8 (2.4–5.5)	4.1 (2.4–6.8)
Years since diagnosis, median (IQR)	9.2 (5.7–15.1)	10.5 (5.4–17.5)	8.8 (5.7–15.7)	9.2 (6.3–13.3)
UC severity prior to surgery, n (%)				
Mild	21 (6 %)	8 (8 %)	8 (6 %)	8 (4 %)
Moderate	50 (14 %)	15 (15 %)	11 (9 %)	24 (19 %)
Severe	280 (80 %)	76 (77 %)	107 (85 %)	97 (77 %)
Number of surgical procedures, n (%)				
2	228 (65 %)	70 (20 %)	100 (28 %)	58 (17 %)
3	90 (26 %)	22 (6 %)	36 (10 %)	32 (9 %)
4	14 (4 %)	5 (1 %)	3 (1 %)	6 (2 %)

### Quality of life and satisfaction with colectomy

The majority of respondents (305, 87 %) reported that they were “somewhat satisfied”, “satisfied”, or “very satisfied” with colectomy. Most (294, 84 %) also reported an improvement in their quality of life post-surgery, with 46 % stating that their current quality of life was “very improved.”

The results of the various survey tools applied are shown in Table 2. The median IBDQ score was 172 (interquartile range 147–190), with clinical remission generally scoring above 170. The mean EQ-5D index score was 0.79 (95 % CI 0.77–0.81) and the mean EQ-5D VAS score was 77.0 (95 % CI 75.3–78.6).

**Table 2:** Survey and scale results of LOCUS participants (n = 351)

Survey	Score: median (IQR), mean ± SD or proportion: n (%)
Inflammatory bowel disease questionnaire	172 (147–190)
EQ-5D	
Utility	0.79 ± 0.2
Visual analog scale	77.0 ± 16.1
Hospital anxiety and depression scale (anxiety scores)	
None (<8)	241 (69 %)
Mild (8–10)	54 (15 %)
Severe (11–21)	52 (15 %)
Missing	4 (1 %)
Hospital anxiety and depression scale (depression scores)	
None (<8)	291 (83 %)
Mild (8–10)	38 (11 %)
Severe (11–21)	20 (6 %)
Missing	2 (1 %)
Body image scale	9 (7–13)
Male	8 (6–11)
Female	11 (8–14)
Age group ≥50 years	8 (6–11)
Age group <50 years	10 (7–13)
Medical outcomes study sexual functioning scale	
Male	8.3 (0–41.7)
Female	19.4 (0–58.3)
Work productivity^a,b^	3.4 ± 2.0 “slightly more productive”
Daily Activity^a,c^	4.2 ± 2.6

^a^Moderate to severe UC patients only

^b^1 = much more productive, 7 = much less productive

^c^Impact of bowel condition on daily activities (other than work/school) in the past month (0–10 scale; 0 = “my condition had no effect on my daily activities”, 10 = “my condition completely prevented me from doing my daily activities”

**Fig. 2:**
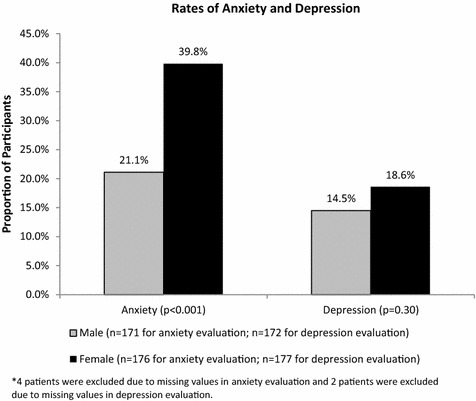
Proportion of LOCUS respondents with anxiety and depression between male and female patients

**Fig. 3:**
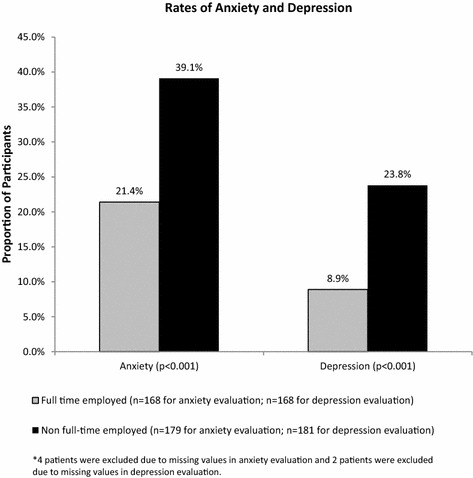
Proportion of LOCUS respondents with anxiety and depression between full-time employed and non-full-time employed patients*

HADS scores identified 30 % of respondents with clinically meaningful anxiety, and 17 % with clinically meaningful depression (scores ≥8 on the anxiety and depression scales indicate the presence of the condition). Women were nearly twice as likely to have clinically meaningful anxiety as men (39.8 vs. 21.1 %, p < 0.001) (Fig. 2). There was no significant difference in clinically meaningful depression scores between male and female patients (18.6 vs. 14.5 %, p = 0.30) (Fig. 2). The results also showed that patients without full-time employment were more likely to have clinically meaningful anxiety and depression than full-time employed patients (anxiety 39.1 vs. 21.4 %, p < 0.001; depression 23.8 vs. 8.9 %, p < 0.001) (Fig. 3).

In response to the BIQ items, 21–34 % of respondents reported that colectomy led to “quite a bit” or “extreme” negative impacts and 34 % reported at least some dissatisfaction with their surgical scar. Body image scale scores were worse among women than men (p < 0.0001) and worse among those <50 years of age (p < 0.05).

MOS-SFS scores varied widely among surveyed UC patients (Table 2). Women reported scores on average 11 points worse than did men. Among moderate to severe UC patients pre-colectomy (n = 330), 27 % of men and 28 % of women reported that their sexual life was worse now than before surgery (Table 3). While only 48 of respondents had tried to conceive since surgery, women were more likely to report having trouble conceiving than men (64 vs. 22 %) (Table 3).

**Table 3:** Fertility questions by gender among participants with moderate to severe ulcerative colitis prior to surgery (N = 330)

	Males	Females
Characteristic	N = 161	N = 169
How has your sexual life changed compared to before surgery?		
Better	27 (17 %)	43 (25 %)
Same	69 (43 %)	42 (25 %)
Worse	44 (27 %)	47 (28 %)
Not applicable or do not wish to answer	21 (13 %)	37 (22 %)
Since surgery		
I have tried to conceive or have biological children	23 (14 %)	25 (15 %)
I have had difficulties conceiving since my surgery^a^	5/23 (22 %)	16/25 (64 %)
Number of children		
None	64 (40 %)	72 (43 %)
1–3	90 (56 %)	72 (54 %)
4 or more	6 (4 %)	4 (2 %)
Do not wish to answer	1 (1 %)	1 (1 %)

Having mild disease prior to surgery is largely uncharacteristic of the pre-colectomy UC population, as colectomy is typically indicated for moderate to severe UC, and thus those patients were excluded from the selected analyses that specifically compared pre- and post-colectomy

^a^Of patients who tried to conceive children

Of all respondents, 68 % were employed either full- or part-time, and 8 % were students. When asked about their current productivity compared to before surgery, moderate to severe patients (n = 312) reported a mean of 3.4 on the WHO Work Performance Questionnaire of survey respondents, corresponding to “slightly more productive”. However, among LOCUS participants with moderate to severe UC prior to surgery, 33 % reported decreased work productivity post-colectomy (Table 4). Overall, respondents reported a mean of 1 day missed from work over the past month due to health reasons, corresponding to approximately 12 days per year on average. A mean score of 4.2, on a scale from 0 to 10, was reported for how UC had affected the ability to perform daily activities (other than work or school) in the past month.

**Table 4:** Report of work productivity, eating restrictions, and stool frequency among participants with moderate to severe ulcerative colitis prior to surgery (N = 330)

Characteristic	N	%
Has your work productivity changed compared to before surgery?		
No change	49	15
Slightly more to much more productive	163	49
Slightly less to much less productive	108	33
Missing	10	3
Have your eating restrictions changed compared to before surgery?		
Fewer	127	39
Same	87	26
More	110	33
Missing	6	2
Has your stool frequency changed compared to before surgery?^a^		
Fewer	157	70
Same	20	9
More	45	20
Missing	2	1

Having mild disease prior to surgery is largely uncharacteristic of the pre-colectomy UC population, as colectomy is typically indicated for moderate to severe UC, and thus those patients were excluded from the selected analyses that specifically compared pre- and post-colectomy

^a^Only patients with an ileal pouch (n = 224, 68 %)

Of the respondents overall, 19 % said that their diet interferes with their daily life “moderately” or “very much”, and 19 % said that they are “moderately” or “very” bothered by the dietary impacts resulting from their surgery for UC. The majority (59 %) of patients with moderate to severe UC prior to surgery experienced the same or more eating restrictions after surgery compared to pre-colectomy (Table 4).

The majority of patients (79 %, 177/224) with an ileal pouch reported improved or stable stool frequency after surgery (Table 4). Although, 46 % (n = 160) of study respondents reported taking medication currently for gastrointestinal conditions, of which the majority (61 %) took medications to regulate bowel movements (Table 5).

**Table 5:** Medication usage for gastrointestinal conditions after surgery among participants

Medication usage reported among LOCUS Participants (N = 160)	%
Medications to regulate bowel movements (loperamide, codeine)	99/160 (61 %)
Antibiotics (ciprofloxacin, metronidazole)	23/160 (14 %)
Immune-modulating agents (thiopurines, anti-TNF monoclonal antibodies, methotrexate)	9/160 (6 %)
Aminosalicylates	7/160 (4 %)
Proton pump inhibitors	5/160 (3 %)
Analgesics (fentanyl, acetaminophen, gabapentin)	5/160 (3 %)
Supplements (iron, B_12_, probiotics)	4/160 (3 %)
Antispasmodics (anti-cholinergics)	4/160 (2 %)
Steroids (rectal steroids, prednisone)	3/160 (2 %)
Other medications for heartburn or nausea	1/160 (2 %)

**Fig. 4:**
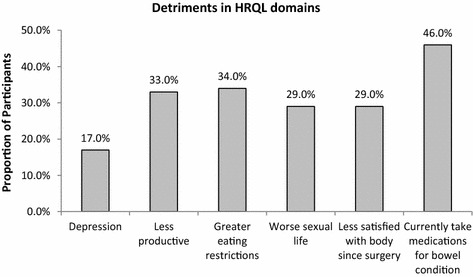
Proportion of LOCUS respondents with detriments in health-related quality of life (HRQL) domains

LOCUS respondents reported detriments in the HRQL domains of mood (i.e., depression) (17 %), work productivity (33 %), diet (i.e., greater eating restrictions) (34 %), sexual life (29 %), body image (29 %), and ongoing need for medication for bowel condition (46 %) (Fig. 4).

## Discussion

Among patients with UC, life after proctocolectomy and IPAA is generally reported to lead to a measurably improved HRQL. While postoperative mortality is low and early post-operative complications have been carefully documented, long term effects on various aspects of HRQL have received less systematic attention. Such knowledge supports therapeutic decision-making for both the doctor and patient. The study attempted to better define such effects by using multiple validated survey tools in a large population of patients with colectomy and either an IPAA or ileostomy (Additional file 1: Table S1 to Table S5).

Not unexpectedly, the majority (84 %) of patients with UC reported an improved HRQL after surgery, as most had poorly controlled disease as the indication for surgery. However, the majority of patients experienced impairments in HRQL in the years following surgery with eight out of ten experiencing detriments in at least one HRQL domain, specifically depression, body image, greater eating restrictions, sexual function, productivity, or ongoing need for medication for bowel condition. A previous study from one institution reported 87 % of 103 UC patients who underwent colectomy were satisfied with the procedure, but 93 % experienced some kind of functional restriction in work, social, physical, or sexual life after surgery (Rokke et al. 2011). The current study showed that such impairments were common regardless of institution or nationality.

About one-third of participants with moderate to severe UC prior to colectomy with IPAA reported they had the same or increased stool frequency after surgery. Additionally, nearly one half of all survey respondents reported taking medications to regulate bowel movements. While all patients had been cured of UC, many patients still scored poorly on the IBDQ, with a score less than the generally-accepted cut-off value representing ‘good’ (>170), as previously described (Lichtenstein et al. 2006; Haapamäki et al. 2009; Meyer et al. 2009; Watanabe et al. 2006).

Overall HRQL is considered a more important measurement of wellbeing after major surgery than any individual symptom score. The EQ-5D is a survey tool demonstrated to have validity for generalizing health status among the general population, persons with different diseases, and across many different countries (Rabin and de Charro 2001; Stark et al. 2010). The EQ-5D has been applied to populations of patients with UC who have not had surgical resection in Germany and Australia. Mean EQ-5D index scores were 0.91 and 0.71 in the German cohort and 0.81 and 0.72 in an Australian cohort for patients with inactive and active disease, respectively (Gibson et al. 2013; Yoshida et al. 2014). Furthermore, in the Australian study, mild disease was associated with a mean EQ-5D score of 0.78 and moderate to severe disease 0.68. In our study, patients with UC post-colectomy reported a mean EQ-5D score of 0.79, suggesting good overall HRQL, but similar to UC patients with mild disease (Stark et al. 2010).

Among LOCUS participants, nearly one in three reported anxiety and one in six reported depression according to the HADS. Depression and anxiety were both more common among those who reported less than full-time employment. These data may reflect that the employment status of the patient may have an impact on the patient’s mood, and provides a potential target for interventional strategies to address in patients with UC post-colectomy.

Approximately one-third of LOCUS participants reported “quite a bit” and “extreme” negative impacts on body image post-colectomy. Not surprisingly, one in three patients had at least some dissatisfaction with the abdominal scar, as most patients had an open laparotomy. One of the key advantages of laparoscopic colectomy and pouch formation is the cosmetic advantage and minimization of wound-related complications such as incisional hernia. It is unlikely, however, that this was a major drive of reduced quality of life. The impact on sexual function was a more likely candidate. Sexual functioning was significantly impacted among UC patients post-colectomy, with more than 25 % of men and women with moderate to severe UC prior to colectomy reporting a worse sexual life after surgery. This is higher than the rate reported from a single centre study conducted in Norway, in which 17 % of study participants reported a worse sexual life post-colectomy (Rokke et al. 2011), although the instruments used to measure such function were not the same. Other studies have reported higher rates of worse sexual function among UC/IBD patients after surgery (Yoshida et al. 2014; Muller et al. 2010). Only 14 % of men and 15 % of women of LOCUS participants with moderate to severe UC prior to surgery had tried to conceive, possibly a reflection of voluntary childlessness previously noted in patients with IBD (Marri et al. 2007), and they described trouble conceiving, with women being more affected.

The findings of this study are also critical for health care payers who strive to achieve cost-effectiveness. Even though surgery may be a cheaper option to pursue for these patients, in the era of personalized care and the increased role of patients in disease management and decision-making, the need for surgery should be evaluated on an individual patient basis. Surgery may not be an acceptable or preferred option for all patients. In their study, to quantify the preferences for treatment options among UC patients, gastroenterologists, and colorectal surgeons, Byrne et al. reported that 89 % of patients, 69 % of gastroenterologists, and 55 % of colorectal surgeons were more prepared to gamble or trade part of their life expectancy to avoid any surgery (Byrne et al. 2014). Bewtra et al. also reported that patients with UC were willing to accept relatively high risk of dying from medical therapy in order to avoid a permanent ostomy (Bewtra et al. 2014). For certain patients, such as patients who have psychological problems, emotional instability, poor motivation or who are non-compliant, the psychological impact of surgical intervention could be immense (Frizelle and Burt 2001). Our study showed that about one-third of participants reported same or increased stool frequency after surgery. In addition, almost one half of all survey respondents reported taking medications to regulate bowel movements. These concerns should also be taken in account, given their significant impact on a patient’s lifestyle and HRQL. In addition, patients with ulcerative proctitis may receive reduced or delayed benefits from surgery compared with those with more extensively-located disease. Therefore, surgery is rarely indicated for this population, while infliximab has shown efficacy in bringing clinical response in patients with ulcerative proctitis (Whitlow 2004; Bouguen et al. 2010). Colectomy may thus not be a “*one size fits all*” solution.

The use of biologics has been recommended by the European Crohn’s and Colitis Organization (ECCO) guidelines and current literature suggests that some of the biologics reduce the need for colectomy (Dignass et al. 2012; Lopez et al. 2015). The use of biologics has also been recommended by the NICE guidelines, “with the choice of treatment between biologics be made on an individual basis after discussion between the responsible clinician and the patient about the advantages and disadvantages of the treatments available” (National Institute for Health and Care Excellence 2015).

The strengths of this study include the array of HRQL domains assessed, which allows for a more comprehensive assessment of HRQL than is currently available in the literature. Further, our recruitment across multiple centers in three countries makes the findings more generalizable to a majority of colectomy patients and meaningful to patients considering colectomy. In addition, patient-reported data were supplemented with medical chart data, which helped to validate and interpret patient-reported data. This study adds to the literature by providing patient-reported information regarding overall HRQL and satisfaction with colectomy.

The data should be interpreted in the context of the study limitations. First, pre-colectomy data were limited to that recorded in the medical chart. Secondly, the study population was captured after surgery and comparison of HRQL measures pre- and post-surgery was not possible. And we do not have a control population that did not undergo the surgery. Thus, the data can only be used to characterize the current status of the study population and provide comparison data with that published in the literature. Thirdly, the response rate was 57 % and, therefore, the findings may be subject to a non-response bias. For example, patients who had extreme perceptions of their surgery (e.g., highly improved or considerably worse off) may have selectively agreed to participate in the study. Fourthly, while validated HRQL measures were used, the data are predominantly based on self-reported information from the patients, and no physician assessment was used to validate the responses. However, this was somewhat minimized by cross-checking of key information by medical chart review, which would have minimized errors associated with, for example, misclassification of diagnosis and surgery.

In conclusion, HRQL is good but not great for UC patients following colectomy, as impairments were found in multiple domains using a variety of assessment tools. While surgical intervention can be lifesaving in many UC patients, induction of medical remission and avoidance of surgery altogether is still the ideal. The data derived from this study provide important information for rational decision-making between physicians and patients, so that the need for surgery may be evaluated on an individual patient basis.

## Additional files

10.1186/s40064-015-1350-7 Additional Tables.
