# Supplementary material for: Long-term outcomes of colectomy surgery among patients with ulcerative colitis
Source: Springerplus. 2015 Oct 5;4:573. doi: 10.1186/s40064-015-1350-7 (PMC4628015; doi:10.1186/s40064-015-1350-7)
Supplement: Supplementary file 1 — 10.1186/s40064-015-1350-7 Additional Tables. [file 40064_2015_1350_MOESM1_ESM.docx]

**Supplementary material**

**Table S1 Sexual functioning and fertility by country**

|  | | | **Country** | | | | | | | | | | | | | | | **All** | | | | | |
| --- | --- | --- | --- | --- | --- | --- | --- | --- | --- | --- | --- | --- | --- | --- | --- | --- | --- | --- | --- | --- | --- | --- | --- |
|  | | | **Australia** | | | **Canada** | | | | | | **UK** | | | | | |  |  |  |  |  |  |
|  | | **N** | | **%** | | **N** | | | **%** | | | **N** | | | **%** | | | **N** | | | | **%** | |
| **How many children do you have?** | | | | | | | | | | | | | | | | | | | | | | | |
| **None** | | | 16 | 44 | | | 60 | | | 41 | | | 67 | | | 45 | | | 143 | | 43 | | |
| **1** | | | 3 | 8 | | | 19 | | | 13 | | | 23 | | | 15 | | | 45 | | 14 | | |
| **2** | | | 12 | 33 | | | 45 | | | 30 | | | 36 | | | 24 | | | 93 | | 28 | | |
| **3** | | | 5 | 14 | | | 22 | | | 15 | | | 15 | | | 10 | | | 42 | | 13 | | |
| **4 or more** | | | 0 | 0 | | | 2 | | | 1 | | | 7 | | | 5 | | | 9 | | 3 | | |
| **Do not wish to answer** | | | 0 | 0 | | | 0 | | | 0 | | | 1 | | | 1 | | | 1 | | 0 | | |
| **missing** | | | 2 | | | | 4 | | | | | | 2 | | | | | | 8 | | | | |
| **Before surgery, was having children (or more children) part of your plan in life?** | | | | | | | | | | | | | | | | | | | | | | | |
| **Yes** | | | 13 | 36 | | | 71 | | | 48 | | | 63 | | | 43 | | | 147 | | 44 | | |
| **No** | | | 18 | 50 | | | 60 | | | 41 | | | 66 | | | 45 | | | 144 | | 44 | | |
| **Unsure** | | | 5 | 14 | | | 15 | | | 10 | | | 17 | | | 11 | | | 37 | | 11 | | |
| **Do not wish to answer** | | | 0 | 0 | | | 1 | | | 1 | | | 2 | | | 1 | | | 3 | | 1 | | |
| **missing** | | | 2 | | | | 5 | | | | | | 3 | | | | | | 10 | | | | |
| **Since you have had your surgery, have you tried to conceive (or have biological children)?** | | | | | | | | | | | | | | | | | | | | | | | |
| **Yes** | | | 4 | 11 | | | 24 | | | 16 | | | 22 | | | 15 | | | 50 | | 15 | | |
| **No** | | | 32 | 89 | | | 121 | | | 82 | | | 124 | | | 84 | | | 277 | | 83 | | |
| **Do not wish to answer** | | | 0 | 0 | | | 3 | | | 2 | | | 2 | | | 1 | | | 5 | | 2 | | |
| **missing** | | | 2 | | | | 4 | | | | | | 3 | | | | | | 9 | | | | |
| **Have you had difficulties conceiving since your surgery? (Among those who have tried to conceive)** | | | | | | | | | | | | | | | | | | | | | | | |
| **Females** | **Yes** | | 2 | | 100 | | | 7 | | | 50 | | | 7 | | | 78 | | | 16 | | | 64 |
|  | **No** | | 0 | | 0 | | | 7 | | | 50 | | | 2 | | | 22 | | | 9 | | | 36 |
| **Males** | **Yes** | | 1 | | 50 | | | 2 | | | 22 | | | 3 | | | 23 | | | 6 | | | 25 |
|  | **No** | | 1 | | 50 | | | 7 | | | 79 | | | 10 | | | 77 | | | 18 | | | 75 |
| **Do not wish to answer** | | | 0 | | | | | 1 | | | | | | 0 | | | | | | 1 | | | |
| **Has your sexual life changed compared with before surgery? *(among patients with moderate-severe UC prior to surgery)*a** | | | | | | | | | | | | | | | | | | | | | | | |
| **Better now** | | | 7 | | 24 | | | 35 | | | 30 | | | 26 | | | 23 | | | 68 | | | 26 |
| **The same** | | | 9 | | 31 | | | 50 | | | 42 | | | 40 | | | 36 | | | 99 | | | 38 |
| **Worse now** | | | 13 | | 45 | | | 33 | | | 28 | | | 46 | | | 41 | | | 92 | | | 36 |
| **Not applicable** | | | 4 | | | | | 15 | | | | | | 21 | | | | | | 40 | | | |
| **Do not wish to answer** | | | 0 | | | | | 6 | | | | | | 9 | | | | | | 15 | | | |
| **missing** | | | 2 | | | | | 3 | | | | | | 2 | | | | | | 7 | | | |

**Table S2 Productivity change by country**

|  | **Country** | | | | | | | **All (N=341)** | | **p-value** |
| --- | --- | --- | --- | --- | --- | --- | --- | --- | --- | --- |
|  | **Australia (n=38)** | | | **Canada (n=152)** | | **Australia (n=38)** | |  |  |  |
|  | **n** | | **%** | **n** | **%** | **n** | **%** | **n** | **%** |  |
| **Has your productivity (what you accomplish) changed since before your surgery for your ulcerative colitis? *(among patients with moderate or severe disease prior to colectomy)*a** | | | | | | | | | |  |
| **Much more productive** | | 4 | 12 | 30 | 22 | 25 | 18 | 59 | 19 | 0.232 |
| **More productive** | | 9 | 26 | 33 | 24 | 38 | 27 | 80 | 26 |  |
| **Slightly more productive** | | 4 | 12 | 9 | 7 | 10 | 7 | 23 | 7 |  |
| **No change** | | 4 | 12 | 26 | 19 | 18 | 13 | 48 | 15 |  |
| **Slightly less productive** | | 4 | 12 | 21 | 15 | 16 | 11 | 41 | 13 |  |
| **Less productive** | | 5 | 15 | 15 | 11 | 18 | 13 | 38 | 12 |  |
| **Much less productive** | | 4 | 12 | 3 | 2 | 16 | 11 | 23 | 7 |  |
| **missing** | | 1 | | 5 | | 3 | | 9 | |  |

^a^Patients with mild disease reported “no change” on average.

**Table S3 Health-Related Absenteeism by Country**

|  | | **Country** | | | | | | | | | | | | | | |  | | | | **p-value** |
| --- | --- | --- | --- | --- | --- | --- | --- | --- | --- | --- | --- | --- | --- | --- | --- | --- | --- | --- | --- | --- | --- |
|  |  | **Australia (n=38)** | | | | | | **Canada (n=152)** | | | | | **UK (n=151)** | | | | **All (N=341)** | | | |  |
|  |  | **n** | | **Mean** | | **SD** | | **n** | | **Mean** | | **SD** | **n** | | **Mean** | **SD** | **n** | **Mean** | **SD** | |  |
| In the past month, please think how much your bowel condition has affected your ability to do your regular daily activities, other than work at a job/be a student. (0=My condition had no effect on my daily activities, 10=My condition completely prevented me from doing my daily activities) | | | | | | | | | | | | | | | | | | | | |  |
|  | 36 | | 3.2 | | 3.1 | | 146 | | 3.0 | | | 2.7 | | 149 | 3.5 | 2.7 | 331 | 3.2 | | 2.8 | 0.196 |
| **WHO-HPQ *Work*** | | | | | | | | | | | | | | | | | | | | | |
| **Days worked per week** | 18 | | 4.7 | | 1.1 | | 107 | | 4.8 | | 1.1 | | | 91 | 4.7 | 0.8 | 216 | 4.7 | | 1.0 | 0.757 |
| **Absolute Absenteeism^a^** | 19 | | 1.0 | | 1.6 | | 107 | | 2.1 | | 3.0 | | | 90 | 2.1 | 2.7 | 216 | 2.0 | | 2.8 | 0.269 |
| **Relative Absenteeism^b^** | 18 | | 0.05 | | 0.1 | | 107 | | 0.11 | | 0.2 | | | 90 | 0.12 | 0.2 | 215 | 0.11 | | 0.2 | 0.267 |
| **Health-related Absolute Absenteeism^c^** | 19 | | 0.4 | | 1.1 | | 107 | | 0.7 | | 1.6 | | | 90 | 0.7 | 1.4 | 216 | 0.7 | | 1.5 | 0.704 |
| **Health-related Relative Absenteeism^d^** | 18 | | 0.02 | | 0.1 | | 107 | | 0.04 | | 0.1 | | | 90 | 0.04 | 0.1 | 215 | 0.04 | | 0.1 | 0.641 |
| **Relative Days of Work^e^** | 18 | | 0.95 | | 0.1 | | 107 | | 0.89 | | 0.2 | | | 90 | 0.88 | 0.2 | 215 | 0.89 | | 0.2 | 0.270 |
| **Absolute Presenteeism^f^** | 19 | | 81.6 | | 13.4 | | 84 | | 81.3 | | 11.4 | | | 89 | 79.8 | 11.4 | 192 | 80.6 | | 11.6 | 0.639 |
| **Relative Presenteeism^g^** | 19 | | 1.1 | | 0.2 | | 84 | | 1.1 | | 0.2 | | | 89 | 1.1 | 0.2 | 192 | 1.1 | | 0.2 | 0.813 |
| **WHO-HPQ *School* ^h^** | | | | | | | | | | | | | | | | | | | | | |
| **Absolute Absenteeism^a^** | 2 | | 5.6 | | 3.0 | | 16 | | 1.6 | | 1.5 | | | 9 | 3.4 | 4.9 | 27 | 2.5 | | 3.2 | 0.151 |
| **Relative Absenteeism^b^** | 2 | | 0.28 | | 0.2 | | 16 | | 0.08 | | 0.1 | | | 9 | 0.17 | 0.2 | 27 | 0.13 | | 0.2 | 0.299 |
| **Health-related Absolute Absenteeism^c^** | 2 | | 0.9 | | 1.2 | | 16 | | 0.3 | | 1.3 | | | 9 | 1.7 | 3.1 | 27 | 0.8 | | 2.1 | 0.151 |
| **Health-related Relative absenteeism^d^** | 2 | | 0.04 | | 0.1 | | 16 | | 0.01 | | 0.1 | | | 9 | 0.08 | 0.2 | 27 | 0.04 | | 0.1 | 0.299 |
| **Relative Days of School^e^** | 2 | | 0.72 | | 0.2 | | 16 | | 0.92 | | 0.1 | | | 9 | 0.83 | 0.2 | 27 | 0.87 | | 0.2 | 0.151 |

^a^Total number of days missed from work/school in the past 28 days for any reason.

^b^Proportion of time missed from work/school in the past 28 days for any reason

^c^Number of days missed from work/school in the past 28 days for health reasons.

^d^Proportion of time missed from work/school in the past 28 days for health reasons.

^e^Proportion of time worked/in school out of total time scheduled to work/be in school

^f^Usual job performance over the past month (0=worst performance, 100=top performance)

^g^Usual job performance over the past month relative to others in similar position (0.5=perform half as well as others, 1.0=same job performance as others, 2=perform twice as well as others)

^h^Students were assumed to attend school 5 days per week.

**Table S4 Survey and scale results of LOCUS participants**

| **Survey** | **Score: median (IQR), mean ± SD or proportion: n (%)** | |
| --- | --- | --- |
|  | **With a stoma** | **Without a stoma** |
| **Inflammatory Bowel Disease Questionnaire** | 163±37 | 168±36 |
| **EQ-5D** |  |  |
| Utility | 0.77± 0.26 | 0.79±0.24 |
| Visual Analog Scale | 75.9 ± 17.5 | 77.0±16.5 |
| **Hospital Anxiety and Depression Scale (Anxiety scores)** |  |  |
| None (< 8) | 68 (67%) | 153(65%) |
| Mild (8-10) | 16 (16%) | 39(17%) |
| Severe (11-21) | 17 (16%) | 35(15%) |
| Missing | 1 (1%) | 7(3%) |
| **Hospital Anxiety and Depression Scale (Depression scores)** |  |  |
| None (< 8) | 81(79%) | 196(84%) |
| Mild (8-10) | 13 (13%) | 23(10%) |
| Severe (11-21) | 7 (7%) | 11(5%) |
| Missing | 1 (1%) | 4(2%) |
| **Body Image Scale** | 11.9±4.4 | 9.4±3.7 |
| Male | 10.7±4.1 | 8.4±3.0 |
| Female | 13.0±4.5 | 10.5±4.0 |
| **Medical Outcomes Study Sexual Functioning Scale** |  |  |
| Male | 34.2±38.3 | 18.1±28.5 |
| Female | 21.0±31.2 | 36.3±35.1 |

**Table S5 Indication for each surgery**

|  | **Country** | | |  |
| --- | --- | --- | --- | --- |
| **Indication** | **Australia(n=38)** | **Canada(n=152)** | **UK(n=151)** | **All (n=351)** |
| **1^st^ surgery** |  | | | |
| **Medically refractory** | **23 (62%)** | **52 (34%)** | **45 (42%)** | **120 (41%)** |
| **Dysplasia** | **5 (10%)** | **5 (4%)** | **5 (5%)** | **15 (5%)** |
| **Complications of colitis** | **15 (40%)** | **57 (36%)** | **12 (11%)** | **84 (28%)** |
| **Other** | **0** | **9(6%)** | **9 (9%)** | **18 (5%)** |
| **Unknown** | **0** | **36 (24%)** | **40 (37%)** | **76 (26%)** |
| **2^nd^ surgery** |  | | | |
| **Medically refractory** | **6 (21%)** | **6 (4%)** | **4 (5%)** | **16 (7%)** |
| **Dysplasia** | **0** | **0** | **0** | **0** |
| **Complications of colitis** | **1 (3%)** | **7 (6%)** | **1 (1%)** | **9 (3%)** |
| **Other** | **20 (69%)** | **97 (73%)** | **51 (69%)** | **169 (71%)** |
| **Unknown** | **2 (7%)** | **22 (17%)** | **21 (28%)** | **45 (19%)** |
| **3^rd^ surgery** |  | | | |
| **Medically refractory** | **1 (11%)** | **1 (2%)** | **0** | **2 (1%)** |
| **Dysplasia** | **0** | **0** | **0** | **0** |
| **Complications of colitis** | **0** | **3 (6%)** | **0** | **3 (3%)** |
| **Other** | **7 (78%)** | **42 (79%)** | **19 (54%)** | **68 (70%)** |
| **Unknown** | **1 (11%)** | **8 (15%)** | **16 (49%)** | **26 (27%)** |
